# Enhanced Nasal Deposition and Anti-Coronavirus Effect of Favipiravir-Loaded Mucoadhesive Chitosan–Alginate Nanoparticles

**DOI:** 10.3390/pharmaceutics14122680

**Published:** 2022-12-01

**Authors:** Khent Primo Alcantara, Nonthaneth Nalinratana, Nopporn Chutiwitoonchai, Agnes L. Castillo, Wijit Banlunara, Opa Vajragupta, Pornchai Rojsitthisak, Pranee Rojsitthisak

**Affiliations:** 1 Center of Excellence in Natural Products for Ageing and Chronic Diseases, Chulalongkorn University, Bangkok 10330, Thailand; 2 Pharmaceutical Sciences and Technology Program, Faculty of Pharmaceutical Sciences, Chulalongkorn University, Bangkok 10330, Thailand; 3 Department of Pharmacology and Physiology, Faculty of Pharmaceutical Sciences, Chulalongkorn University, Bangkok 10330, Thailand; 4 National Center for Genetic Engineering and Biotechnology (BIOTEC), National Science and Technology Development Agency, Pathum Thani 12120, Thailand; 5 Faculty of Pharmacy, The Graduate School, Research Center for the Natural and Applied Sciences (RCNAS), University of Santo Tomas, Manila 1008, Philippines; 6 Department of Pathology, Faculty of Veterinary Science, Chulalongkorn University, Bangkok 10330, Thailand; 7 Molecular Probes for Imaging Research Network, Faculty of Pharmaceutical Sciences, Chulalongkorn University, Bangkok 10330, Thailand; 8 Department of Food and Pharmaceutical Chemistry, Faculty of Pharmaceutical Sciences, Chulalongkorn University, Bangkok 10330, Thailand; 9 Metallurgy and Materials Science Research Institute, Chulalongkorn University, Bangkok 10330, Thailand

**Keywords:** response surface methodology, Box–Behnken design, chitosan, alginate, SARS-CoV-2, antiviral, polymeric nanoparticles, favipiravir, intra-nasal

## Abstract

Favipiravir (FVR) is a repurposed antiviral drug for treating mild to moderate cases of the novel coronavirus disease 2019 (COVID-19). However, its poor solubility and permeability limit its clinical efficacy. To overcome its physicochemical and pharmacokinetic limitations, we statistically designed a mucoadhesive chitosan–alginate nanoparticles (MCS-ALG-NPs) as a new carrier for FVR using response surface methodology, which provided suitable characteristics for transmucosal delivery. The use of mucoadhesive polymers for intranasal administration promotes the residence time and contact of FVR in the mucus membrane. The optimized FVR-MCS-ALG-NPs demonstrated superior mucoadhesion, higher permeation and deposition in the nasal mucosa, and a significant increase in the inhibition of viral replication over 35-fold compared with free FVR. The overall results suggest that MCS-ALG-NPs could be used as an effective mucoadhesive carrier to enhance the activity of FVR against COVID-19.

## 1. Introduction

The novel coronavirus disease 2019 (COVID-19) remains a primary global health concern due to its high infectivity and mortality rate. Its causative agent, severe acute respiratory syndrome coronavirus 2 (SARS-CoV-2) commonly spreads through the inhalation of airborne droplets or direct contact with the virus in the mucosa [1]. Current strategies to mitigate the spread of SARS-CoV-2 focus on prevention through social distancing, wearing masks, and the investigation of vaccines. However, the efficacy of vaccines has been in question due to recurring mutations in the viral genome [2], and the efficacy of the existing vaccines needs to be re-evaluated for new SARS-CoV-2 variants. Because of these variants, contingency measures including drug development with optimal efficacy towards mutant stains become as equally important as vaccine development. As new drug candidates for COVID-19 are still on their way, repurposed small-molecule antivirals are being utilized for COVID-19 treatment including protease inhibitors (lopinavir/ritonavir) and RNA-dependent RNA polymerase (RdRp) inhibitors (remdesivir, molnupiravir, and favipiravir (FVR)) [3]. Fortunately, patients infected with other COVID-19 variants still respond to RdRp inhibitors since the mutations happen mainly in the spike protein of SARS-CoV-2 and not in the translation of viral polymerase protein or RdRp [4]. Further, reports on repurposed RdRp inhibitors (e.g., FVR, remdesivir, molnupiravir) for COVID-19 were found to have low retention at the site of infection, non-specificity and low absorption and bioavailability owing to their poor solubility and their hydrophobic macromolecule structures [5,6]. To improve the solubility and permeability of FVR using an ionic-liquid-based formulation which provides a 1.5-fold increase in the oral absorption and peak blood concentration of FVR was reported [7]. A nanotechnology approach has been employed to overcome these physicochemical limitations of FVR, including the use of lipid nanoparticles (lipid-NPs) and nanoemulsions which improved not only its solubility but also its activity against SARS-CoV-2 in vitro [8,9]. This improvement in the anti-viral activity of nanomaterial-based drug delivery for COVID-19 was hypothesized to facilitate the binding to viral RdRp, thus preventing SARS-CoV-2 replication and subsequently destroying the viral structure [8].

To further improve medication efficacy and targeted drug delivery, the administration of therapeutics through the nose, which is also the primary transmission route of SARS-CoV-2, appears to be suitable for achieving high localization of antivirals. Intranasal delivery has already been used in various respiratory viral infections as it provides either local or systemic effects proximal to infection. It also offers several advantages over systemic delivery systems because of its non-invasiveness, fast onset of action, and in various cases can reduce the side effects of the drug through its direct and targeted delivery approach [10]. In addition, earlier reports on COVID-19 revealed that the nasal cavities and nasopharynx harbor a significant amount of localized SARS-CoV-2, both in symptomatic and presymptomatic carriers [1]. Several studies have been conducted for topical intranasal delivery of antiviral therapies for respiratory infections, particularly COVID-19 [11]. This includes the intranasal delivery of virucidal agents, such as hydrogen peroxide [12], povidone-iodine [13], and buffered saline [11], which demonstrated the reduction of viral load in the upper airway. The delivery of interferons and probiotics through this route was well tolerated and effective as prophylaxis. However, the nasal mucosa remains challenging because of its rapid clearance, mucus entrapment, and low deposition in the nasal epithelium [10].

Thus, the development of NPs using mucoadhesive biopolymers can effectively overcome these mucosal barriers and provide high drug deposition in the nasal cavities. Mucoadhesion emerged as a strategy that promotes sustained release, improved residence time via their interaction with the mucus for immobilization, and enhanced site-specificity and cellular uptake [14]. Positively charged polymers, such as chitosan (CS), have been used for transmucosal delivery of either polar or non-polar drugs due to their various mucoadhesive mechanisms through electrostatic interaction, hydrogen bonding, and hydrophobic interaction with mucus. CS could transiently and reversibly open the tight junctions of the epithelial cells, thereby enhancing the paracellular permeability [15]. In addition, its inherent pH and enzyme sensitivity by protonation of amino groups at acidic pH and the hydrolysis of *β*-(1-4) glycosidic bonds, respectively, made it more favorable for nasal physiological conditions (pH 5.5 and the presence of lysozyme at ~30 µg/mL) [16]. Pyrc et al. demonstrated that CS had an anti-SARS-CoV-2 property through its electrostatic binding to the virus, which prevents viral entry into host cells [17]. However, issues with physical stability, low encapsulation efficiency, and drug loading of hydrophobic molecules are often observed in CS-NPs. On the other hand, alginate (ALG) is a negatively charged polymer that has been used to fabricate NPs to enhance the bioavailability of drugs [18]. ALG has been shown to have antiviral properties against various viruses through their interactions with the viral envelope [19].

In theory, direct local therapies to the upper respiratory tract where the highest viral load could be found may provide an alternative way to potentially reduce the viral loads and transmission of SARS-CoV-2. However, data on the encapsulation of antiviral agents, especially FVR, and their nasal delivery for respiratory infections are limited. In a previous study, the possible mechanism associated with anti-SARS-CoV-2 infection of antiviral agent-loaded nanoformulation was postulated as the prevention of viral adsorption and invasion into the host cell through its interference with the viral envelope, leading to cellular uptake enhancement and eventually viral replication inhibition by targeting the RdRp. As such, we anticipate that the MCS-ALG-NPs can improve the physicochemical properties of FVR and effectively inhibit SARS-CoV-2 replication. The mucoadhesive property of the carrier also permits the transmucosal permeation and deposition of FVR in the nasal mucosa to directly target localized viruses in the upper airway. To achieve the desired characteristics of the FVR-MCS-ALG-NPs for the transmucosal delivery, this study used Box–Behnken design (BBD) and response surface methodology (RSM). Mucoadhesive, in vitro release and ex vivo transmucosal studies of optimized FVR-MCS-ALG-NPs were also performed. Finally, its antiviral activity against porcine epidemic diarrhea virus (PEDV) was evaluated. Herein, we demonstrated a well-tolerated and efficient mucoadhesive and muco-penetrating nanocarrier for FVR with a high permeation rate and deposition in the nasal mucosa while subsequently enhancing its anti-coronavirus effect by 35-fold.

## 2. Materials and Methods

### 2.1. Chemicals and Materials

Favipiravir (FVR, anhydrous, >98% purity) was purchased from Aurore Pharmaceuticals (Telangana, India). Chitosan (CS, Molecular weight (MW) of 73 kDa, degree of deacetylation of 91.7%, and polydispersity index (PDI) of 3.8) was supplied by Marine Bio-Resources (Samut Sakorn, Thailand). Sodium alginate (ALG, low viscosity, A1112), Poloxamer-407, and porcine mucin (type III) were purchased from Sigma-Aldrich, Inc. (St. Louis, MO, USA). All chemicals used in this study were analytical grade without further purification.

### 2.2. Fabrication and Optimization by BBD and RSM

The FVR-MCS-ALG-NPs were fabricated using emulsification and ionotropic gelation method with some modifications [20]. The methanolic FVR solution at various concentrations (5, 10 and 15 mg/mL) was added dropwise to an ALG solution (4 mL, 0.6 mg/mL in ultrapure water, pH 5.5) containing Poloxamer-407 at various concentrations (1,1.5 and 2% *w*/*v*). The solution was magnetically stirred at 1500 rpm for 20 min followed by sonication to allow FVR to completely emulsify. Then, an aqueous CaCl_2_ solution (4 mL, 0.6 mg/mL) was added dropwise at a rate of 20 mL/h using an automatic syringe pump. After 30 min of stirring, the CS solution (4 mL, at various mass ratios to ALG at 0.025, 0.0625 and 1.00; pH 5.5) was added to the pre-gelled calcium–ALG solution and continuously stirred for another 30 min. The suspension was equilibrated overnight in a light-proof cabinet at ambient room temperature. To optimize the fabrication process and determine the effect of various factors on the size (*Y*_1_), ζ-potential (*Y*_2_), loading capacity (LC) (*Y*_3_), and encapsulation efficiency (EE) (*Y*_4_) of the FVR-MCS-ALG-NPs, a three-factor BBD was used based on the preliminary experimental data gathered considering the ALG:CS (*A*), FVR (*B*), and poloxamer 407 (*C*) as the independent variables (Table 1). A total of 15 formulation conditions were generated using Design-Expert^®^ (Stat-Ease^®^ Inc., Minneapolis, MN, USA) with three replicated center points to limit the error (Table 2).

### 2.3. Physicochemical Characterization

The size and PDI of the NPs were measured directly by dynamic light scattering (DLS), and the ζ-potential was determined by electrophoretic mobility of the NPs, using a Zetasizer (Nano-ZS (Malvern Panalytical Ltd., Malvern, UK) without further dilution or pH adjustments (pH 5.5 ± 0.3). The morphology was observed by TEM (JEM-1400Flash, JEOL Ltd., Tokyo, Japan) and FE-SEM (JSM-7610F, Oxford X-Max 20, JEOL Ltd., Tokyo, Japan). LC and EE of the FVR-MCS-ALG-NPs were determined using an indirect method after ultracentrifugation at 4 °C and 105,000 × g speed for 1 h. The absorbance was measured at 363 nm, and the amount of the free FVR from the supernatant was calculated against the standard curve (Agilent Cary 60, Agilent Technology, Santa Clara, CA, USA). The LC (Equation (1)) and EE (Equation (2)) of the FVR-MCS-ALG-NPs were calculated as follows:

LC (%) = [(Wt − Ws)/Wnp)] × 100(1)

EE (%) = [(Wt − Ws)/Wt)] × 100(2)

where Wt is the initial amount of FVR added in the formulation, Ws is free FVR in the supernatant, and Wnp is the weight of the NPs after lyophilization.

The thermal properties were determined using a thermogravimetric analyzer (TG 209 F3 Tarsus^®^, Netzsch, Germany) in a nitrogen atmosphere at 20 °C/min. The crystalline structure was analyzed using a wide-angle X-ray diffractometer (XRD, PANalytical X’Pert Pro model, Kassel-Waldau, Germany), operated at a speed of 0.2° 2θ/step at room temperature.

### 2.4. Mucoadhesion Study

The size and ζ-potential were measured to observe their interactions with mucin [21]. A mucin solution (0.4 mg/mL) was prepared in phosphate-buffered solution (PBS, pH 7.4) with 5% mucin. An equal amount of mucin and the FVR-MCS-ALG-NP suspension was shaken in an incubator shaker set at 150 rpm and 37 °C. After ultracentrifugation of the incubated mixture, the precipitate was washed with water to remove unbound mucin. The resuspended FVR-MCS-ALG-NPs were analyzed for the change in size and ζ-potential using a Zetasizer (Nano-ZS). The mucin:water mixture was used as the control for the analysis.

#### In Vitro Mucin Adsorption Studies

The adsorption and interaction between the NPs and mucin were evaluated based on the published methods [22]. The unbound mucin in the supernatant from Section 2.4 was quantified by bicinchoninic acid (BCA) protein analysis. The absorbance of the mixture was measured at 562 nm, and the amount of the mucin was calculated against its standard curve. The mucin binding efficiency was computed using Equation (3).

Mucin binding efficiency (%) = [(Mt − Ms)/Mt)] × 100(3)

where Mt is the total amount of mucin initially added, Ms is unbound mucin from the supernatant.

### 2.5. In Vitro FVR Release Study

Ready-to-use SnakeSkin™ dialysis tubing (Thermo Scientific, Decatur, IL, USA) was used as a membrane for the study. The sealed tubes were then submerged in 500 mL of PBS (pH 7.4) and simulated nasal fluid (SNF) (8.77 mg/mL NaCl, 2.98 mg/mL KCl and 0.59 mg/mL CaCl_2_ in deionized water, pH 5.5) to mimic the mucus layer and nasal epithelium environment, respectively. The medium was maintained at 37 °C and stirred at 100 rpm. At pre-determined time points, an aliquot was withdrawn and centrifuged at 4000 rpm. The amount of free FVR from the supernatant was calculated against the standard curve of FVR. The cumulative FVR released was computed using Equation (4).

(4) $$CR\;\left(\%\right)=\frac{V_{e}\;\sum_{i=1}^{n-1}C_{n-1}+V_{o\;}C_{n}}{m}\times 100$$

where *CR* (%) is cumulative FVR released, *V_e_* is collection volume (mL), *V_o_* is the volume of the medium (mL), *C_n_* is the concentration of the time point (mg/mL), and m is total FVR (mg) in the dialysis bag.

### 2.6. Ex Vivo Transmucosal Studies

Porcine nasal mucosa (PNM) was collected from the noses of freshly slaughtered pigs in the local slaughterhouse and was processed as described in supplementary materials S1.1. SNF was used as the medium for the Franz cell diffusion experiment. The PNM was pre-saturated with SNF onto the donor compartment for 15 min. After equilibration, 1 mL of FVR-MCS-ALG-NPs or free FVR solution (457 µg of FVR) was placed in the donor compartment. The receptor chamber was maintained at 37 ± 0.5 °C and stirred at 150 rpm. An aliquot was withdrawn and filtered before quantification for FVR. The cumulative amount of FVR permeated per unit area (µg/cm^2^) was plotted as a function of time (min). After the experiment, the FVR retained in the donor compartment was quantified. The flux at steady state (Jss) and permeability coefficient (K_P_) were estimated using Equations (5) and (6), respectively.

Jss = ΔQt/(Δt × A)(5)

K_P_ = Jss/C_0_ (6)

where ΔQt (µg) is the difference in the permeated drug amount between the time points (Δt (h)), A (cm^2^) is the diffusion area, and C_0_ (µg) is the initial drug concentration in the donor compartment. To determine the effect on the permeability compared to the free FVR solution, the enhancement ratio of the flux (ER_flux_) was computed using Equation (7):

ER_flux_ = Jss_FVR-NPs_/Jss_free FVR_ (7)

### 2.7. Biocompatibility Studies

The biocompatibility studies were performed in human nasal cells and porcine tissue. RPMI 2650 human nasal epithelial cells (ATCC, Manassas, VA, USA) were cultivated in MEM media supplemented with 10% (*v*/*v*) fetal bovine serum (FBS) at 37 °C in a 95% air/5% CO_2_ incubator. The diluents were used as the control to observe the cytotoxic effect of the test samples.

The histopathological examination of the treated and untreated PNM from Section 2.6 was performed after 1 h of exposure to treatment to determine any possible mucosal damage. PNM was fixed in 10% buffered formalin immediately after the experiment. The tissues were embedded in paraffin and processed accordingly for sectioning. The 4 µm thickness of the sectioned tissues was stained with routine hematoxylin and eosin and analyzed by a veterinary pathologist.

### 2.8. Viral Infectivity

The mCherry-PEDV was added to the seeded eGFP-Vero cells to allow viral absorption for 1 h. The unattached virus was removed by washing with PBS. The test samples were added to the cells with fresh media supplemented with 1% tryPLE. Sterile water or blank NP-treated cells (0.5% *v*/*v*) were used as the control to observe the antiviral effect of the test samples. At 18 h post-infection, the mCherry (561_ex_ and 570–630_em_ nm) and eGFP (488_ex_ and 500–550_em_ nm) fluorescent images were obtained using the Opera Phenix High-Content Screening System (PerkinElmer Inc., Waltham, MA, USA). The total mCherry fluorescent intensity was quantified by Harmony high-content imaging and analytical software (PerkinElmer Inc., Waltham, MA, USA). The relative decrease in mCherry intensity of the test samples was calculated by comparison with the control.

### 2.9. Statistical Analysis

The experiments are expressed as mean ± standard deviation (SD). The half-maximal effective concentration (EC_50_) was determined through a non-linear regression-curve-fit analysis. The difference between groups was compared by ANOVA followed by a postoperative multiple comparison test in GraphPad^®^ Prism software 8.3.0. (San Diego, CA, USA) A *p* value of <0.05 was considered significant.

## 3. Results

### 3.1. Analysis and Selection of Optimal Mathematical Model

In this study, we focused on studying the effects and interaction of crucial factors in developing CS-based NPs, particularly the CS, FVR, and poloxamer concentrations to produce a stable system with suitable characteristics for nano-based drug delivery. Previous studies discussed the influence of ALG:CS mass ratio in size and encapsulation efficiency (EE) owing to the low electrostatic affinity of the polymers leading to instability of the system. Furthermore, the effects of the drug concentration, especially hydrophobic molecules, along with the surfactant concentration play an important role in the formulation [23]. This is due to its effect on the size and % EE due to the production of unstable droplets and high tendency of coalescence because of unbalanced surface tension on the particles’ surfaces [23]. Preliminary experiments were conducted, and the concentration range being studied for the independent variables was selected based on previously published literature that gave a suitable physical characteristic for a nano-based drug delivery system and a stable formulation of hydrophobic drugs.

All 15 formulations were prepared successfully as a clear solution without any visible signs of separation or precipitation prior to characterization. The summary of the experimental runs and their corresponding responses are shown in Table 2 as the average from three independent batches. The recorded responses were fitted to different mathematical models (linear, quadratic, and 2-factor interaction) in Design Expert^®^ software (Version 13, Stat-Ease Inc., Minneapolis, MN, USA) to find a suitable model for optimization. ANOVA was utilized in this study to compare the variation resulting from the change in the combination of variable levels and the variation brought by the random errors in the measurements of the generated responses. The result of the statistical analysis of the mathematical model is summarized in Table S1 in Supplementary. The program automatically chose the optimal model based on the following criteria: (1) the model must be significant, (2) the lack-of-fit must not be significant, (3) the adjusted R^2^ and predicted R^2^ must be high and in reasonable agreement (<0.2 difference), and (4) the selected model must have high adequate precision (>4 is acceptable).

**Figure 1:**
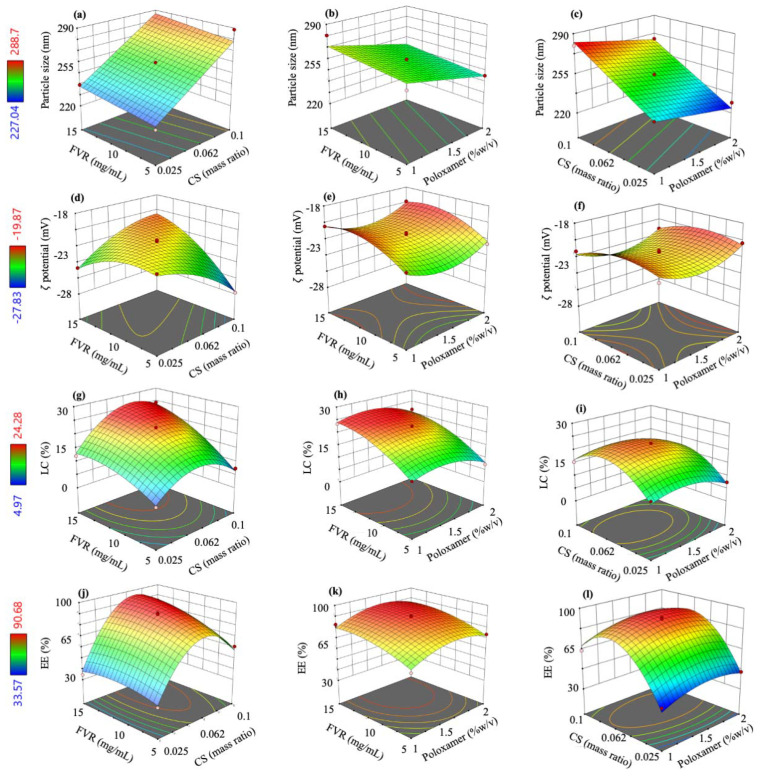
Three-dimensional (3D) surface model plots showing the interaction between the independent factors to (**a**–**c**) size, (**d**–**f**) ζ potential, (**g**–**i**) LC, and (**j**–**l**) EE.

The fit summary of the responses and the selected model are presented in Table S2 in the Supplementary. Moreover, polynomial equations were generated for each of the responses (Equations (8)–(11)) and were used to identify the relative impact of each factor. Note that the (+) sign of the coefficients indicates a positive impact or a synergistic effect between the variables, while (−) coefficients denote otherwise. From these coded equations, three-dimensional (3D) response surface plots were illustrated to show the effects of the investigated independent factors on dependent responses (Figure 1).

*Y*_1_ = 258.29 + 24.35*A* + 4.08*B* − 8.95*C* (8)

*Y*_2_ = −21.51 − 0.3213*A* + 1.13*B* + 0.2362*C* + 2.46*AB* − 1.03*A*^2^ − 1.25*B*^2^ + 1.42*C*^2^ (9)

*Y*_3_ = 22.13 + 2.90*A* + 6.23*B* − 1.74*C* + 2.5*AB* − 7.19*A*^2^ − 2.76*B*^2^ − 3.37*C*^2^ (10)

*Y*_4_ = 89.19 + 16.86*A* + 6.81*B* + 3.26*C* + 5.81*AB* − 30.96*A*^2^ − 4.80*B*^2^ − 8.42*C*^2^ (11)

### 3.2. Optimization and Validation

The optimal conditions were computed based on searches for a combination of factor levels that would satisfy the desired goals for each response and independent factor. The goal for each factor and response are shown in Table 1. The value with the highest desirability function of 0.93 was selected as the optimized condition for the formulation (Table 3). The optimal conditions were prepared in six replicates, and the responses were assessed to validate the model. The observed experimental values for each response must closely agree with the predicted responses computed by the software to render the model valid. The predicted and observed responses are reported in Table 3 with minimal % error demonstrating the validity and robustness of the model selected and the computed optimal variables.

### 3.3. Characterization of Optimized FVR-MCS-ALG-NPs

#### 3.3.1. Physicochemical Characterization

**Figure 2:**
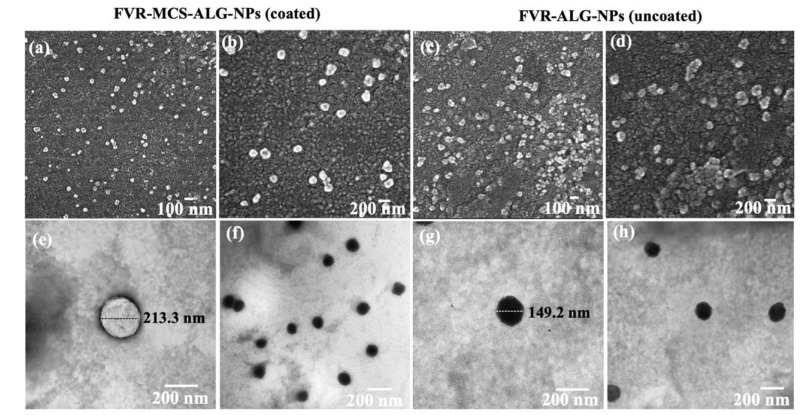

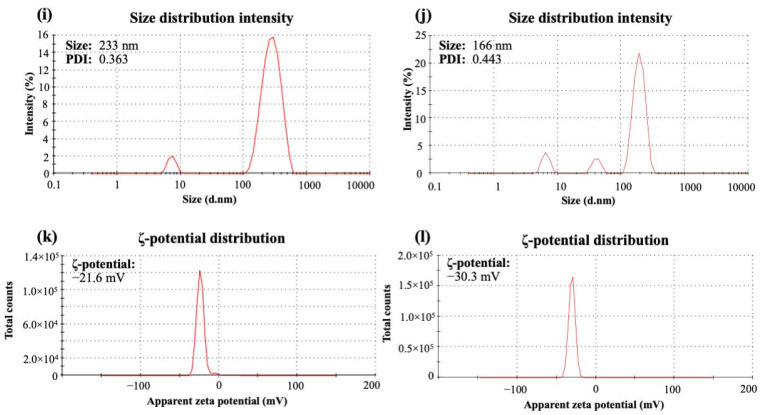
Characteristics of the optimized NPs: SEM images (at 50,000× (**a**,**c**) and 100,000× (**b**,**d**) magnification) and TEM images (100,000× (**e**,**g**) and 50,000× (**f**,**h**) magnifications); size distribution and ζ-potential of the optimal FVR-MCS-ALG NPs (**i**,**k**) and uncoated FVR-ALG NPs (**j**,**l**).

Smaller particles have a higher surface area that is advantageous for absorption and transport across the nasal cavity. The SEM (Figure 2a–d) and TEM (Figure 2e–h) images of FVR-MCS-ALG-NPs and uncoated FVR-ALG-NPs demonstrated well-dispersed spherical particles with minimal aggregation. As presented in Figure 2i–l, the uncoated-FVR-ALG-NPs exhibited a relatively small size and a higher (−)-charge than the FVR-MCS-ALG-NPs due to the absence of positively charged CS on the surface. A high LC (26.0 ± 0.7) and EE (84.6 ± 0.7) were achieved in the optimal formulation, which was beneficial for obtaining a high drug concentration in the polymer matrix while subsequently reducing the dosage concentration.

Additionally, thermal analyses of FVR showed only a single drop at 238.0 °C attributed to the decomposition of FVR. Furthermore, three-step weight losses were observed in the TGA/DTG curve of the blank-MCS-ALG-NPs (Figure S1a,b in Supplementary) due to the loss of bound water, formation of Na_2_CO_3_, and the total decomposition of the polymer [24]. The same thermogram was observed for the FVR-MCS-ALG-NPs with an additional loss at 322.9 °C due to degradation of residual FVR. The XRD patterns of FVR as shown in Figure S1c (see Supplementary) were consistent with previous studies, confirming the crystalline structure and the purity of the compound [25]. Broad peaks at 19.3° and 23.5° 2θ were observed in both blank-MCS-ALG-NPs and FVR-MCS-ALG-NPs, associated with the amorphous pattern of poloxamer 407. Furthermore, the absence of the crystalline FVR in the FVR-MCS-ALG-NPs was observed as ascribed to its amorphous dispersion in the system following encapsulation in MCS-ALG-NPs.

#### 3.3.2. Mucoadhesive Study of FVR-MCS-ALG-NPs

**Figure 3:**
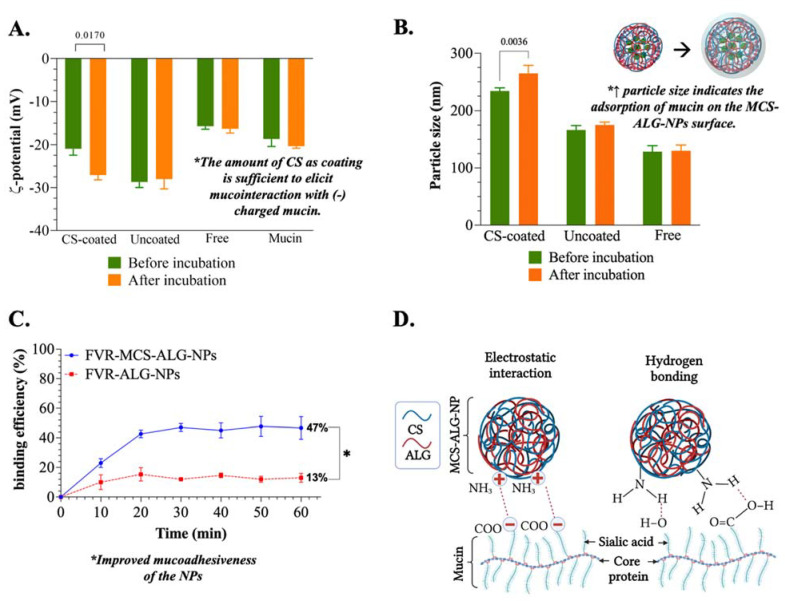
Mucoadhesive study: (**A**) ζ-potential and (**B**) size of the samples before and after incubation with mucin for 1 h; (**C**) mucin binding efficiency of the FVR-MCS-ALG-NPs and FVR-ALG-NPs as a function of time, *n* = 9 (* *p* = 0.0073); (**D**) illustration of the proposed mechanism of mucoadhesion of FVR-MCS-ALG-NPs.

CS and ALG were chosen as the materials to construct the NPs owing to their different mucoadhesion mechanisms. A non-ionic mucoadhesive poloxamer was also chosen to stabilize the system due to its absorption-enhancing effects in the nasal epithelium. The electrostatic interaction is one of the most common and expected mechanisms of mucoadhesion. The CS-coated FVR-ALG-NPs (or FVR-MCS-ALG-NPs) changed significantly in ζ-potential (*p* = 0.0170) after 1 h incubation with mucin (Figure 3A) suggesting the interaction of the CS coating of the FVR-ALG-NPs with the sialic acid of the mucin. Because of this, it can be noted that the size of CS-coated FVR-ALG-NPs also significantly increased (*p* = 0.0036) owing to the adsorption of mucin on the NP surface (Figure 3B). Moreover, the uncoated-FVR-ALG-NPs remained unchanged relative to the pre-incubated samples and the mucin control. This indicates that electrostatic interaction is not present in the system demonstrated by its inability to interact with mucin and alter its surface charge and particle size.

The % mucin binding onto the NPs was also investigated to better understand its mucoadhesive properties. Significant mucin binding was observed in the first 10 min of incubation, wherein FVR-MCS-ALG-NPs (23.0 ± 7.0%) had more significant mucin adsorption compared to the FVR-ALG-NPs (10.0 ± 4.4%) (*p* < 0.0001) (Figure 3C). After 1 h, the FVR-MCS-ALG-NPs had a % binding efficiency of 46.8 ± 9.1%, higher than the FVR-ALG-NPs with only 13.0 ± 3.1% mucin adsorbed (*p* < 0.0001). The statistical analysis revealed no significant change in the % of mucin adsorbed after 10 min to 1 h incubation of FVR-ALG-NP samples indicating saturation of mucin binding on the NPs. Moreover, the % mucin binding in the FVR-MCS-ALG-NPs showed a gradual increase up to 30 min of incubation.

#### 3.3.3. In Vitro Release Study of FVR from FVR-MCS-ALG-NPs Using SnakeSkin™ Artificial Membrane

**Figure 4:**
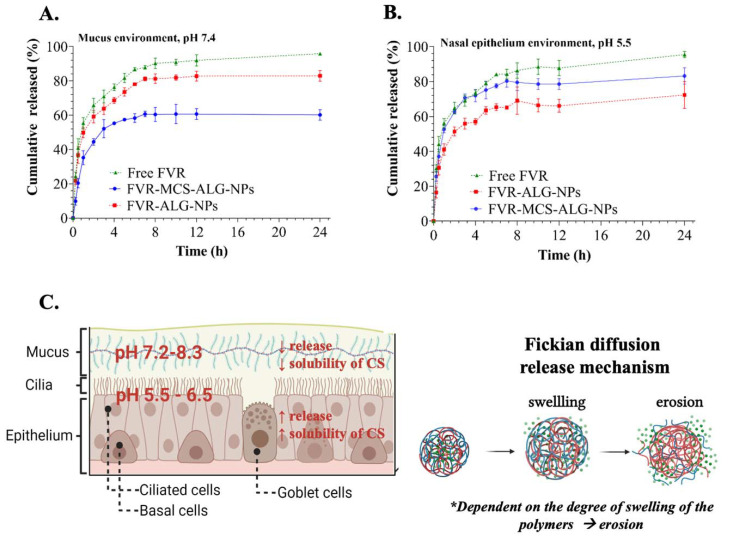
In vitro FVR released from NPs in the (**A**) mucus and (**B**) nasal epithelium environment (*n* = 3) through SnakeSkin™ artificial membrane; (**C**) illustration of the proposed release mechanism of FVR from the MCS-ALG-NPs.

Formulations with and without CS were compared to observe the benefit of a CS coating on the release of FVR from the NPs (Figure 4A,B). The release behavior of NPs in the mucus (pH 7.4) and nasal epithelium (pH 5.5) environments was studied. A 60.2 ± 2.5% (CS-coated) and 83.0 ± 2.5% (uncoated) of the FVR were released from the NPs within 24 h at mucus environment (pH 7.4), and a total of 83.3 ± 3.8% (CS-coated) and 72.2 ± 6.5% (uncoated) under the SNF or epithelium environment (pH 5.5). Notably, the CS coating greatly affected the release of FVR considering the pH of the environment which favors the acidic nasal mucosa environment, suggesting its suitability for nasal administration.

To evaluate the release kinetics of the samples, the release data were fitted to various models including zero-order, first-order, Higuchi, Korsmeyer–Peppas, Hixson–Crowell, and Weibull using the DDsolver program [26]. The best model was chosen based on the following criteria: (1) the R_2_ value must be close to 1, (2) the model selection criteria (MSC) must be high, and (3) the Akaike information criterion (AIC) must be low [27]. The summary of the release kinetics using various models is presented in Tables S3–S5 of the Supplementary. Based on the criteria mentioned, all experimental samples best fit the Weibull model in both media. The fit of the release data was further analyzed based on Weibulls’ two geometric parameters (α and β). The α is a scale parameter defining the time scale of the process. While the β is a shape parameter characterizing the curve shape as exponential (β = 1), sigmoidal or S-shaped, with upward curvature followed by a turning point (β > 1), or parabolic, with a higher initial slope and then consistent with an exponential (β < 1) [28]. The analysis of the computed β of the release data (β: 0.24–0.36) did not correlate with the criteria of the curve shape as parabolic (β > 1) since the plotted release data demonstrated an exponential shape of release depicting that the model may not be appropriate to explain the release kinetics. Hence, Korsmeyer–Peppas or the diffusion/relaxation model being the second-best model was used to predict the release kinetics of the NPs. The FVR release transport mechanism was characterized based on the computed “*n*” value: (1) *n* ≤ 0.5, the drug diffusion from the polymer matrix corresponds to a Fickian diffusion or a quasi-Fickian diffusion mechanism. (2) 0.5 < *n* < 1, an anomalous, non-Fickian drug diffusion occurs. (3) *n* = 1, a non-Fickian, case of II (relaxational) transport or zero-order release kinetics could be observed, and (4) *n* > 1, super case II transport was observed [29]. The computed “*n*” value from 0.19 to 0.23 depicts a Fickian diffusion mechanism of FVR release from the degradation of the polymer matrix (Figure 4C) [29]. Hoang Thai et al. explained that the release of CS–ALG NPs involves a combination of mechanisms including the swelling and the dissolution of the polymers and/or the diffusion and dissolution of the drug [30,31,32]. This study also complies with the previous investigations of CS-based NPs release suggesting that the release kinetics of the drug from the NPs is primarily governed by the swelling of the polymer, drug diffusion through the matrix, polymer erosion, and the combination of erosion and degradation [30,33,34].

### 3.4. Nasal Mucosa Permeation and Retention Studies

**Figure 5:**
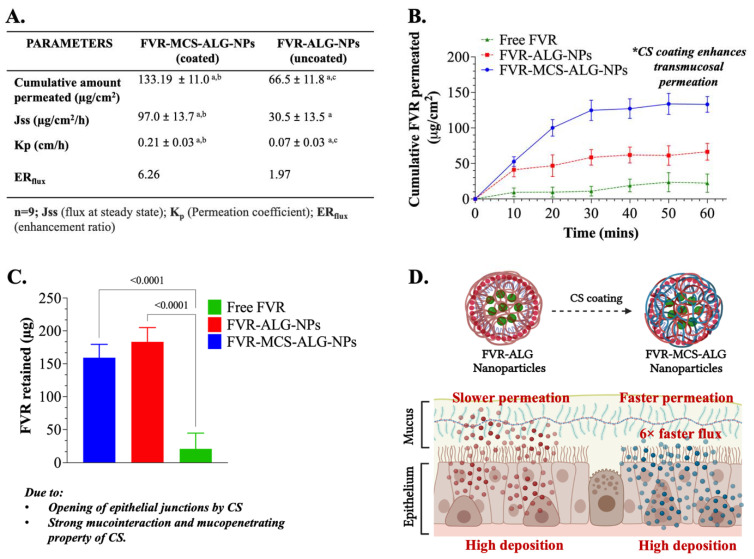
(**A**) Comparison of the permeation profiles of FVR-MCS-ALG-NPs and FVR-ALG-NPs through PNM. The same letter within each row indicates a statistical significance at *p* < 0.05.; (**B**) permeability study of FVR through PNM; (**C**) approximate amount of FVR deposited in the PNM after 1 h; (*n* = 9); (**D**) illustration of the permeation and deposition properties of the NPs.

The transport and retention of FVR through PNM were investigated in nine (9) mucosal specimens from each treatment group. FVR-MCS-ALG-NPs (133.2 ± 11.0 µg/cm^2^) had superior permeation through the membrane with an area of 1.77 cm^2^ compared to the uncoated FVR-ALG-NPs (66.5 ± 11.8 µg/cm^2^) and free FVR (22.3 ± 13.8 µg/cm^2^) (Figure 5A,B). Due to the small size of the NPs and larger surface area, it can be noted that both NP formulations had a significant permeation after 1 h than the free FVR (*p* < 0.0001). The FVR-MCS-ALG-NPs had a significant permeation activity through PNM compared to the FVR-ALG-NPs (*p* < 0.0001). In addition, a significant amount of the FVR from the FVR-MCS-ALG-NPs and FVR-ALG-NPs was deposited in tissues compared to free FVR. Interestingly, the FVR retained (*p* = 0.0834) was similar to both coated and uncoated FVR-NPs. This can be explained by the slower permeation of the uncoated FVR-ALG-NPs that causes the deposition of FVR in the mucosa (Figure 5A,C). The superior permeation profile is evident in the computed Jss and K_p_ for the FVR-MCS-ALG-NPs which was significantly higher than the uncoated FVR-ALG-NPs and free FVR. The ER_flux_ values showed 6-fold and 3-fold enhanced drug flux for FVR-MCS-ALG-NPs and uncoated FVR-ALG-NPs, respectively, versus free FVR.

### 3.5. Biocompatibility Study in RMPI 2650 Human Nasal Epithelia and Porcine Nasal Mucosa

**Figure 6:**
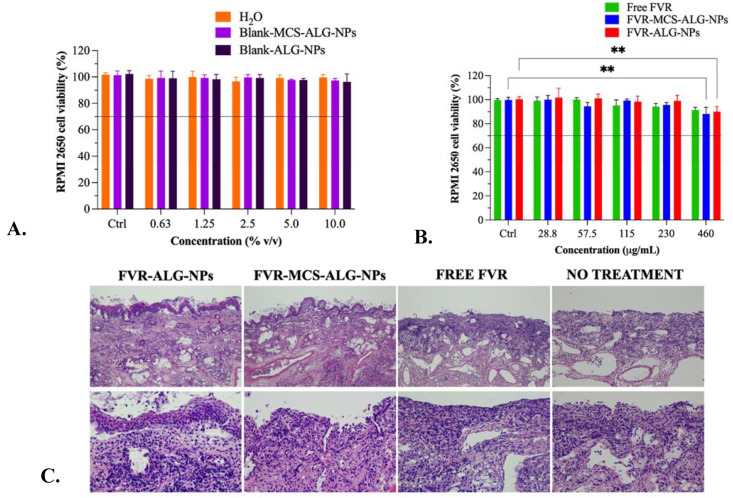
(**A**,**B**) Cytocompatibility study of NPs in RPMI 2650 human nasal epithelium, (*n* = 3 independent experiments). The control cells containing media without any treatments were used to observe the cytotoxic effect of the test samples. The line signifies the lower limit for safety assessment (>70% viability) based on the ISO 10993-5 criteria [38]. ** *p* < 0.001 compared to the control; (**C**) Histopathology of porcine nasal mucosa shows no significant lesions after 1 h of exposure to treatments viewed under low (top row) and high (bottom row) magnifications.

Charged polymers such as CS and ALG could exert cytotoxicity when they aggregate onto the cell surface and interferes with intracellular activities; hence, cytocompatibility of the nanocarrier must be established [35]. In this study, we investigated the cytocompatibility of the NP formulation towards the RPMI 2650 cell line, which is the only immortalized human nasal epithelial cell that is well-studied for in vitro testing of products that are intended for nasal delivery [36,37]. The toxicity of the water and blank-NPs as a diluent for free FVR and formulated FVR-NPs (FVR-MCS-ALG-NPs and FVR-ALG-NPs) were first investigated. Results revealed that the diluents did not cause any significant toxicity to the cell even at its highest concentration; hence 10% *v*/*v* of the diluent was normalized as the final concentration for the dilution in subsequent experiments (Figure 6A).

Sequentially, the cytotoxicity of the free FVR and FVR-NPs serially diluted in water and blank-NPs in various concentrations were investigated. There was a significant difference in the cell viability of RPMI 2650 when treated with the highest working concentration of 460 µg/mL (>88% viability) for FVR-NPs as compared to the control cells (Figure 6B). It is also evident that the reduction in cell viability is attributed to the drug itself as the control which was treated as the diluents used did not have any substantial cytotoxicity. Additionally, the cytotoxicity of FVR-NPs could be from the enhanced cellular uptake of the NPs in the RPMI 2650. Nonetheless, these values still comply with the ISO 10993-5 criteria (Biological evaluation of medical devices Part 5: Test for in vitro cytotoxicity), wherein it says that the reduction in cell viability by >30% is considered cytotoxic [38]. Hence, it can be deduced that the free FVR and FVR-MCS-ALG NPs have excellent compatibility with RPMI 2650. Moreover, the histopathological changes in the excised nasal mucosa were examined by a licensed veterinary pathologist who was blinded to the treatment. The nasal mucosal histology showed no significant lesions, which are common indications of toxicity in nasal epithelium by NPs (Figure 6C).

### 3.6. Antiviral Activity of FVR against PEDV

**Figure 7:**
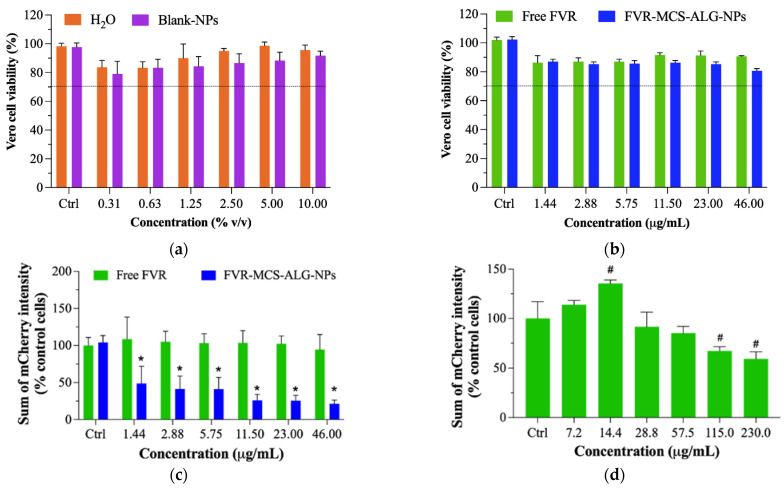

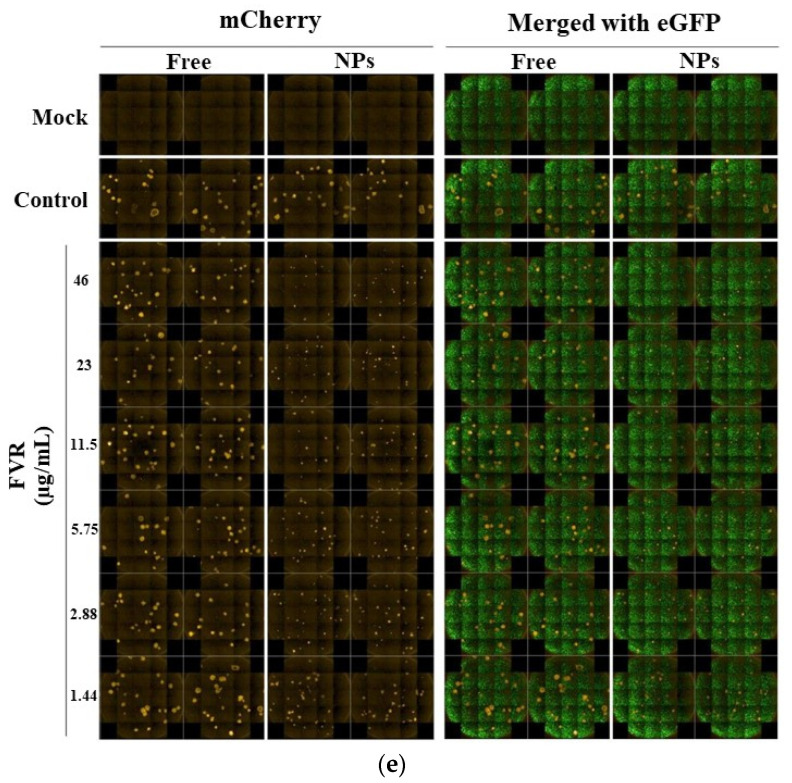
Vero cell viability: (**a**) diluents and (**b**) FVR (formulated and unformulated) after 18 h. The line signifies the lower limit for safety assessment (>70% viability) based on the ISO 10993-5 criteria [38]; Anti-coronavirus effect: (**c**) Average quantitative mCherry fluorescence intensity data of the infected cells from three independent experiments (*n* = 3), (**d**) anti-coronavirus effect of free FVR in higher concentrations, (**e**) The stitched images of mCherry and GFP signals. # *p* < 0.05 and * *p* < 0.0001 compared to the control.

As SARS-CoV-2 is classified as a risk group 3 pathogen that requires extremely stringent safety precautions and specialized laboratory facilities, we employed non-infectious coronavirus as a surrogate for the antiviral studies. The porcine epidemic diarrhea virus (PEDV, NCBI accession LC053455) was selected as a model due to its low health risk and minimal laboratory requirements. It has biophysical properties and genomic structures similar to human coronaviruses, such as SARS-CoV-2 [39]. Previous reports on the proof-of-concept of the use of the PEDV corona model have proven it to be a reliable surrogate for SARS-CoV-2 [40]. Vero cells stably expressing eGFP (eGFP-Vero) were used as host cells for PEDV carrying the mCherry fluorescent reporter gene (mCherry-PEDV) (Supplementary materials S1.2). The cytotoxic effect of sterile water (H_2_O) and blank-MCS-ALG-NPs was first tested to determine the possible toxicity of the diluent in eGFP-Vero cells as described in Supplementary materials S1.3. Neither test sample exhibited any relative cytotoxicity to eGFP-Vero cells even at the maximum concentration (Figure 7a and Figure S2). Furthermore, the non-cytotoxic concentrations of FVR showed no substantial toxicity to host cells (>80% viability) across all concentrations investigated (Figure 7b).

The anti-viral effect of FVR samples was performed in three independent experiments (Figure S5 in Supplementary). The stitched images of mCherry and eGFP signals from the cells were acquired by a high-content imaging system at 21 images/well (Figure 7e, Figures S3 and S4 in Supplementary). The antiviral assay of FVR-MCS-ALG-NPs caused a drastic reduction in viral replication with an EC_50_ of 6.63 ± 2.42 μg/mL calculated by GraphPad^®^ Prism software 8.3.0 (Figure 7c). The post hoc test confirmed that the FVR-MCS-ALG-NPs had a significant inhibitory effect on viral replication even at its very lowest working concentration compared to free FVR and the control cells (*p* < 0.0001), while free FVR did not have any significant reduction in PEDV replication up to 46.0 μg/mL (*p* > 0.9). To further determine the antiviral activity of free FVR, we investigated the samples in higher concentrations that were non-toxic to the eGFP-Vero cells (Figure 7d and Figure S6 in Supplementary). We found that the free FVR had a significant reduction in viral replication starting at a concentration of 115 μg/mL. These results strongly suggest that the FVR-MCS-ALG-NPs exhibited a significant improvement in antiviral activity towards coronavirus by more than 35-fold compared to the free drug (EC_50_ 6.63 ± 2.42 vs. >230 μg/mL).

## 4. Discussion

Several strategies have been employed to overcome the poor solubility and permeability of FVR from chemical synthesis to formulation design. Nanotechnology using solid-lipid NPs (SLN) has proven to enhance the antiviral effect of FVR towards SARS-CoV-2. Still, issues about the physical characteristics of the fabricated SLN and the instability of the lipid overtime call for the need to explore another suitable carrier for the drug. In this study, we introduced the concept of using polymers as a biomaterial in constructing a nanocarrier for hydrophobic drugs owing to their tunable properties concerning size, surface charge, LC, and EE that are imperative in the delivery, efficacy, and stability of the product. We also demonstrated the concept of using the intranasal route of delivering FVR as it mimics the natural transmission of the SARS-CoV-2 virus. The use of natural biocompatible carbohydrate polymers, such as CS and ALG, as the materials for the construction of nanocarriers could be a rational approach for producing an efficient and stable carrier. Moreover, their tunable size, surface characteristics, and mucoadhesive properties make them suitable for transmucosal delivery. The proposed delivery route for FVR could potentially reduce the viral load in the infected individuals and could eventually mitigate the risk of viral transmission.

Here, we first demonstrate the optimization of the FVR-MCS-ALG-NPs by statistical analysis using RSM based on BBD of experiments in developing an ideal NP formulation for transmucosal delivery. The model employed had high predictability for the critical parameters with the highest desirability of 93% with respect to the goal set for the size, surface charge, LC, and EE. In investigating the effects and interaction between the major factors and responses, we found that the CS mass ratio to ALG majorly contributes to the optimization of FVR-MCS-ALG-NPs. Increasing the concentration of CS in the system contributes to the formation of larger particles brought by the presence of excess CS molecules causing the clumping and entanglement of the molecules [41]. CS also contributes to the LC and EE of the optimized NPs attributed to the increase in amino groups that interact and cross-link with the COOH of ALG [23]. Further, no chemical interaction was observed in the FVR-MCS-ALG-NPs by thermal analysis but the absence of the crystalline FVR in the XRD patterns of FVR-MCS-ALG-NPs was observed which is attributed to the amorphous dispersion of FVR within the MCS-ALG-NPs during the intermolecular interaction of polymers [42].

Additionally, we investigated the benefit of CS-coating onto the NPs regarding drug release, mucoadhesive, and transmucosal permeation by comparing it with the uncoated-NPs (FVR-ALG-NPs) and free FVR. Here, we demonstrated sustained release of the FVR-MCS-ALG-NPs in both pH conditions (7.4 mucus environment and 5.5 nasal epithelium). Notably, the MCS-ALG-NPs had a higher release rate in the epithelium environment than in the mucus, owing to the pH-responsive drug release mechanism of CS than the uncoated-NPs. The amino groups of CS shrink at basic pH due to its deprotonation causing a slow release of FVR at pH 7.4. In contrast, an acidic pH below the isoelectric point (pH 6.5) of CS triggers the protonation of the NH_2_^+^, followed by the swelling and erosion of the polymer matrix and, eventually, the release of FVR [43].

Finding the correct balance between the critical factors in optimization, especially the concentration of the CS-coating, is important to maintain its ideal physical characteristics without compromising the mucoadhesive property of the NPs by electrostatic interaction with the mucus. The investigation of the mucoadhesive property of the NPs confirmed that the optimal amount of CS-coating on the FVR-ALG-NPs is still sufficient to elicit muco-interaction which is evident in the significant change in the size and surface charge of the NPs. The oligosaccharide chain of the mucin presents a negatively charged sialic acid terminal residue that enrobes its interaction with the CS layer, hence the change in the ζ-potential to be more negative. This demonstrated that even though the FVR-MCS-ALG-NPs have a negative surface charge after CS-coating, the optimal concentration of CS can still obtain mucoadhesion that is driven by electrostatic interaction with the positively charged amine groups of D-glucosamine molecule of CS with the sialic acid residue of mucin [44].

Moreover, we also demonstrated the sufficiency of the CS-coating to exhibit enhanced transmucosal permeation through PNM. Our result showed that the optimal concentration of CS on FVR-ALG-NPs still demonstrates a 3- and 6-fold faster flux and superior deposition in the PNM compared to uncoated-NPs and free FVR. In other studies, favorable transmucosal permeation is associated with CS through its ability to disrupt tight epithelial junctions [15]. However, this rationale generally supports the permeation of hydrophilic drugs, and some evidence has proved that it is less efficient to open tight junctions when used as a coating than as the main material in constructing the NPs [44,45]. Here, we postulated that the higher permeation and deposition of the FVR-MCS-ALG-NPs could be explained by a combination of factors specifically its small size and proven mucoadhesive property that propels the particle to move through the mucus layer and facilitate its absorption into the nasal epithelium.

Additionally, we have proven the enhancement of the antiviral effect of the FVR-MCS-ALG-NPs by more than 35-fold compared to free FVR towards the PEDV model that acts as a surrogate virus for SARS-CoV-2. A published concept of improving the antiviral activity of FVR by nano-emulsion for coronavirus was hypothesized to effectively interfere with viral adsorption, invasion, and replication [8]. Another study demonstrated the use of SLN as a carrier for FVR, which resulted in a significant decrease in the EC_50_ of FVR [9]. Comparing the antiviral activity observed in this current study of using polymeric nanocarrier for FVR delivery, we have demonstrated superior enhancement of antiviral activity compared to previous reports. This can be explained by the collective features of the optimal formulation owing to its small size and high drug loading, its mucoadhesive and muco-penetrating property, and the enhanced permeation and deposition of FVR in the nasal mucosa. These collective properties of the FVR-MCS-ALG-NPs could have increased the intracellular drug concentration due to improved cellular uptake while subsequently boosting its antiviral activity.

## 5. Conclusions

This study confirmed that FVR could be delivered via intranasal administration to inhibit coronavirus replication, and its activities could be enhanced by encapsulation in MCS-ALG-NPs. The results demonstrated successful optimization of the physical characteristics of FVR-MCS-ALG-NPs by employing BBD-RSM. The optimal FVR-MCS-ALG-NPs notably exhibited high permeation and deposition of FVR in the nasal mucosa which further explains the enhancement of the antiviral activity of FVR in the PEDV coronavirus model. Finally, we have developed an efficient mucoadhesive NP carrier as a new delivery strategy for FVR through intranasal administration in targeting and reducing coronavirus replication in the upper airways.

## Figures and Tables

**Table 1:** Investigated variables used in BBD.

Variables	Levels	
	Low	High
*Independent* (*Factors*)		
*A* = ALG:CS mass ratio	1:0.0250	1:0.1000
*B* = FVR (mg/mL)	5	15
*C* = Poloxamer-407 (% *w*/*v*)	1	2
*Dependent* (*Responses*)	*Goals*	
*Y*_1_ = size (nm)	Minimize	
*Y*_2_ = ζ-potential (mV)	≥ ± 20	
*Y*_3_ = LC (%)	Maximize	
*Y*_4_ = EE (%)	Maximize	

**Table 2:** BBD experimental matrix and response values for the optimization of FVR-MCS-ALG-NPs.

Code	*A*	*B*	*C*	*Y*_1_ (nm)	*Y*_2_ (mV)	*Y*_3_ (%)	*Y*_4_ (%)
F1	1: 0.0250	5	1.5	227 ± 6	−21.9 ± 0.6	5.0 ± 0.9	33.7 ± 5.6
F2	1: 0.1000	5	1.5	289 ± 16	−27.8 ± 0.4	7.4 ± 0.5	60.9 ± 5.0
F3	1: 0.0250	15	1.5	239 ± 2	−24.7 ± 0.5	12.1 ± 0.9	34.4 ± 3.5
F4	1: 0.1000	15	1.5	284 ± 5	−20.8 ± 0.6	24.3 ± 0.3	84.8 ± 0.7
F5	1: 0.0250	10	1	243 ± 7	−21.6 ± 0.9	11.4 ± 0.5	33.6 ± 0.3
F6	1: 0.1000	10	1	288 ± 5	−21.3 ± 2.0	15.4 ± 0.8	58.9 ± 0.8
F7	1: 0.0250	10	2	231 ± 8	−20.3 ± 0.3	7.4 ± 0.6	37.4 ± 3.8
F8	1: 0.1000	10	2	274 ± 14	−21.2 ± 1.1	12.0 ± 0.6	69.4 ± 0.9
F9	1: 0.0625	5	1	258 ± 16	−22.5 ± 1.2	11.9 ± 0.6	63.3 ± 0.3
F10	1: 0.0625	15	1	281 ± 8	−20.4 ± 0.7	23.4 ± 1.1	82.8 ± 2.9
F11	1: 0.0625	5	2	245 ± 16	−22.6 ± 2.3	7.3 ± 0.5	73.7 ± 4.3
F12	1: 0.0625	15	2	248 ± 12	−19.9 ± 1.0	21.5 ± 1.9	84.2 ± 3.3
F13 *	1: 0.0625	10	1.5	258 ± 5	−21.4 ± 0.7	22.0 ± 0.8	90.7 ± 1.0
F14 *	1: 0.0625	10	1.5	260 ± 7	−22.0 ± 2.8	22.6 ± 2.9	87.1 ± 13.3
F15 *	1: 0.0625	10	1.5	251 ± 14	−21.2 ± 3.2	21.9 ± 0.9	89.8 ± 0.8

(*) are the three replicated center points of the design.

**Table 3:** The compositions and values of optimal conditions and their predicted and observed responses.

Optimal Conditions	Responses	Predicted Responses	Observed Responses	% Error
*A*: 1: 0.057 (ALG:CS mass ratio)	*Y*_1_ (nm)	261.8	233.5 ± 7.7	–10.7
*B*: 12.871 (mg/mL)	*Y*_2_ (mV)	–21.2	–21.6 ± 0.8	1.8
*C*: 1.24 (% *w*/*v*)	*Y*_3_ (%)	24.0	26.0 ± 0.7	8.5
	*Y*_4_ (%)	84.1	84.6 ± 0.7	0.6

% Error was computed as (observed − predicted/predicted) × 100; (*n* = 6).

## Data Availability

Not applicable.

## Supplementary Materials

The following supporting information can be downloaded at: https://www.mdpi.com/article/10.3390/pharmaceutics14122680/s1, S1.1 Collection and preparation of tissue; S1.2 Cell and virus preparation (Reference [46] is cited in the Supplementary Materials); S1.3 Cytotoxicity assay; Table S1: Statistical analysis of the mathematical model; Table S2: Fit summary and the selected mathematical model of the responses; Table S3: Release kinetics of free FVR solution; Table S4: Release kinetic of FVR from MCS-ALG-NPs; Table S5: Release kinetic of FVR from ALG-NPs; Figure S1: Characteristics of optimized FVR-MCS-ALG-NPs comparing with the excipients: (a) TGA, (b) DTG thermal curves and (c) XRD pattern; Figure S2: Cytotoxicity of the FVR-MCS-ALGP-NPs. eGFP-Vero cells were treated with H2O or NPs at the indicated concentrations for 18 h; Figure S3: Antiviral effect of FVR (formulated and unformulated) from experiment 2; Figure S4. Antiviral effect of FVR (formulated and unformulated) from experiment 3; Figure S5: Quantitative data of the antiviral effect of FVR (formulated and unformulated) from 3 independent experiments; Figure S6: Antiviral effect of free FVR at high concentrations.
