# Supplementary material for: Enhanced Nasal Deposition and Anti-Coronavirus Effect of Favipiravir-Loaded Mucoadhesive Chitosan–Alginate Nanoparticles
Source: Pharmaceutics. 2022 Dec 1;14(12):2680. doi: 10.3390/pharmaceutics14122680 (PMC9782217; doi:10.3390/pharmaceutics14122680)
Supplement: Supplementary file 1 [file pharmaceutics-14-02680-s001.zip › pharmaceutics-1983403-supplementary.pdf]

pharmaceutics

MDPI

Enhanced Nasal Deposition and Anti-coronavirus Effect of Favipiravir-Loaded Mucoadhesive Chitosan-Alginate Nanoparticles

Khent Primo Alcantara, Nonthaneth Nalinratana, Nopporn Chutiwitoonchai, Agnes L. Castillo, Wijit Banlunara, Opa Vajragupta, Pornchai Rojsitthisak, Pranee Rojsitthisak

S1. SUPPLEMENTARY METHODS

S1.1 Collection and preparation of the tissue

Porcine nasal mucosa (PNM) was excised from the noses of freshly slaughtered pigs in the local slaughterhouse. The noses were cross-sectioned using a pathology saw through each nostril to expose the tissue. The mucosa was excised using a surgical blade and forceps to separate the underlying nasal septal cartilage. The PNM was washed in normal saline solution (NSS) and submerged in Hanks' solution to maintain tissue integrity.

S1.2 Cell and virus preparation

RPMI 2650 human nasal epithelial cells (ATCC, USA) were cultivated in MEM media supplemented with 10% (v/v) fetal bovine serum (FBS) at 37 °C in a 95% air/5% CO_2 incubator. PEDV carrying the mCherry fluorescent reporter gene (mCherry-PEDV) in its genome was used as a coronavirus surrogate for the antiviral assay. The viral genome was constructed by reverse genetics, and the infectious viral particles were prepared as reported previously [1]. Vero cells stably expressing eGFP, a green fluorescent protein (eGFP-Vero), were constructed by transfecting the pEGFP-N1 (Clontech, USA) plasmid into Vero cells (ATCC: CCL-81) and selecting the eGFP positive cells using 0.8 mg/mL of G418 antibiotic (Sigma Aldrich, USA). The cells were maintained in Opti-MEM (Gibgo, USA) supplemented with 10% (v/v) fetal bovine serum and antibiotics.

S1.3 Cytotoxicity assay

The eGFP-Vero cells were seeded overnight at a density of 2.5×10^4 in a 96-well tissue culture microplate at 37 °C in a 5% CO_2 incubator. The formulation and free FVR were diluted in sterile water or blank-MCS-ALG-NPs and added to the cells at various concentrations. The sterile water-treated cells or the blank MCS-ALG-NPs were used as the control to observe the cytotoxic effects of the test samples. After 18 h of treatment, the cytotoxicity was determined by adding Cell Counting Kit-8 (CCK-8) reagent (Dojindo Molecular Technologies, USA) to the cells and incubating for 1 h. The optical density at OD_{450} was measured using an Ensight Multimode Plate Reader (PerkinElmer, USA).

Table S1 Statistical analysis of the mathematical model

	Y_1	Y_2	Y_3	Y_4
**ANOVA for the model**				
Sum of squares	5517.47	54.10	660.74	6554.42
Degree of freedom	3	7	7	7
Mean squares	1839.16	7.73	94.39	9.36.35
F-value	53.21	45.22	91.11	69.37
p-value	<0.0001	<0.0001	<0.0001	<0.0001
Inference	significant	significant	significant	significant
**Lack-of-fit test**				
Sum of squares	335.37	0.8690	6.95	87.52
Degree of freedom	9	5	5	5
Mean squares	37.26	0.1738	1.39	17.50
F-value	1.66	1.06	9.24	5.03
p-value	0.4312	0.5502	0.1005	0.1741
Inference	not significant	not significant	not significant	not significant
**Residual**				
Sum of squares	380.18	1.20	7.25	94.48
Degree of freedom	11	7	7	7
Mean squares	34.56	0.1790	1.04	13.50

Table S2 Fit summary and the selected mathematical model of the responses

Model	Sequential *p*-value	Lack of Fit *p*-value	Adjusted R^2	Predicted R^2	Adequate precision	Remarks
**Response: Y_1**						
Linear	< 0.0001	0.4312	0.918	0.8729	21.9385	Suggested
2FI	0.143	0.5328	0.9407	0.8653		
Quadratic	0.8048	0.3743	0.9208	0.6516		
**Response: Y_2**						
Linear	0.4431	0.0332	− 0.0073	− 0.6778		
2FI	0.0726	0.0504	0.394	− 0.7722		
Quadratic	0.0006	0.5854	0.9627	0.8681	25.6039	Suggested
**Response: Y_3**						
Linear	0.0147	0.0051	0.4922	0.371		
2FI	0.8341	0.0037	0.3695	0.0293		
Quadratic	0.0002	0.0829	0.9775	0.8776	24.8412	Suggested
**Response: Y_4**						
Linear	0.1088	0.008	0.2499	− 0.0122		
2FI	0.947	0.0056	0.0123	− 0.9547		
Quadratic	< 0.0001	0.1604	0.9734	0.8621	22.1788	Suggested

Table S3 Release kinetics of free FVR solution

Model	Media	Parameter	Adjusted R^2	AIC	MSC
Zero-order ($F = k_0 \cdot t$)	SNF	$K_0 = 6.96$	− 1.49	142.81	− 1.72
	pH 7.4	$K_0 = 7.14$	− 1.25	143.41	− 1.52
First-order ($F = 100 \cdot e^{-k_1 t}$)	SNF	$k_1 = 0.48$	0.76	109.77	0.64
	pH 7.4	$k_1 = 0.52$	0.87	103.46	1.34
Higuchi ($F = k_H \cdot t^{0.5}$)	SNF	$K_H = 28.55$	0.41	122.52	− 0.27
	pH 7.4	$K_H = 29.29$	0.48	122.72	− 0.04
Korsmeyer-Peppas ($F = k_{KP} \cdot t^n$)	SNF	$k_{KP} = 54.32$, n= 0.237	0.97	83.16	2.54
	pH 7.4	$k_{KP} = 53.80$, n= 0.22	0.94	94.28	1.90
Hixson-Crowell ($F = 100 \cdot [1 - (1 - k_{HC} \cdot t)^3]$)	SNF	$k_{HC} = 0.07$	0.39	123.03	− 0.31
	pH 7.4	$k_{HC} = 0.07$	0.50	122.21	− 0.01
[a]Weibull ($F = 100 . [1 - e^{-(t-T1)\beta/\alpha}]$)	SNF	$\alpha = 1.551$, $\beta = 0.369$, $T_i = 0.187$	0.9894	65.1715	3.8132
	pH 7.4	$\alpha = 1.23$, $\beta = 0.46$, $T_i = 0.14$	0.99	62.76	4.24

[a]Best fit release kinetic model for free FVR. *F*: fraction (%) of drug released in time *t*; k_0: zero-order release constant; k_1: first-order release constant; k_H: Higuchi release constant; k_{KP}: release constant incorporating structural and geometric characteristics of the drug-dosage form; *n*: diffusional exponent indicating the drug release mechanism; α: scale parameter defining the time scale of the process; β is shape parameter characterizing the curve shape as exponential (β = 1), sigmoid or S-shaped, with upward curvature followed by a turning point (β > 1), or parabolic, with a higher initial slope and then consistent with an exponential (β < 1) [2]; T_i: location parameter representing the lag time before the onset of the dissolution or release process, which in most cases will be near zero; k_{HC} is the Hixon-Crowell release constant. The transport mechanism based on the "n" value of Korsmeyer-Peppas is described as: (1) n ≤ 0.5, the drug diffusion from the polymer matrix corresponds to a Fickian diffusion or a quasi-Fickian diffusion mechanism. (2) 0.5 < n < 1, an anomalous, non-Fickian drug diffusion occurs. (3) n = 1, a non-Fickian, case of II (relaxational) transport or zero-order release kinetics could be observed, and (4) n >1, super case II transport was observed [3].

Table S4 Release kinetic of FVR from MCS-ALG-NPs

Model	Media	Parameter	Adjusted R^2	AIC	MSC
Zero-order	SNF	$K_0 = 6.32$	− 1.68	141.84	− 1.78
($F = k_0 \cdot t$)	pH 7.4	$K_0 = 4.72$	− 1.05	133.17	− 1.30
First-order	SNF	$k_1 = 0.37$	0.64	113.87	0.21
($F = 100 \cdot e^{-k_1 t}$)	pH 7.4	$k_1 = 0.14$	0.35	117.09	− 0.15
Higuchi	SNF	$K_H = 26.24$	0.30	123.04	− 0.44
($F = k_H \cdot t^{0.5}$)	pH 7.4	$K_H = 19.55$	0.48	113.84	0.08
Korsmeyer-Peppas	SNF	$k_{KP} = 51.34$,	0.92	93.30	1.68
($F = k_{KP} \cdot t^n$)		n= 0.19			
	pH 7.4	$k_{KP} = 53.21$,	0.94	93.20	
		n= 0.21			
Hixson-Crowell	SNF	$k_{HC} = 0.06$	0.29	123.21	− 0.45
($F = 100 \cdot [1 - (1 – k_{HC} \cdot t)^3]$)	pH 7.4	$k_{HC} = 0.04$	0.1822	120.33	− 0.39
[a]Weibull	SNF	$\alpha = 1.23$,	0.98	72.05	3.20
($F = 100 . [1 – e^{-(t-T1)\beta/\alpha}]$)		$\beta = 0.31$,			
		$T_i = 0.22$			
	pH 7.4	$\alpha = 1.89$,	0.95	82.56	2.31
		$\beta = 0.24$,			
		$T_i = 0.33$			

[a]Best fit release kinetic model for free FVR. *F*: fraction (%) of drug released in time *t*; k_0: zero-order release constant; k_1: first-order release constant; k_H: Higuchi release constant; k_{KP}: release constant incorporating structural and geometric characteristics of the drug-dosage form; *n*: diffusional exponent indicating the drug release mechanism; α: scale parameter defining the time scale of the process; β is shape parameter characterizing the curve shape as exponential ($\beta = 1$), sigmoid or S-shaped, with upward curvature followed by a turning point ($\beta > 1$), or parabolic, with a higher initial slope and then consistent with an exponential ($\beta < 1$) [2]; T_i: location parameter representing the lag time before the onset of the dissolution or release process, which in most cases will be near zero; k_{HC} is the Hixon-Crowell release constant. The transport mechanism based on the "n" value of Korsmeyer-Peppas is described as: (1) $n \leq 0.5$, the drug diffusion from the polymer matrix corresponds to a Fickian diffusion or a quasi-Fickian diffusion mechanism. (2) $0.5 < n < 1$, an anomalous, non-Fickian drug diffusion occurs. (3) $n = 1$, a non-Fickian, case of II (relaxational) transport or zero-order release kinetics could be observed, and (4) $n > 1$, super case II transport was observed [3].

Table S5 Release kinetic of FVR from ALG-NPs

Model	Media	Parameter	Adjusted R^2	AIC	MSC
Zero order	SNF	$K_0 = 5.34$	− 1.26	135.74	− 1.51
($F = k_0 \cdot t$)	pH 7.4	$K_0 = 6.36$	− 1.34	140.87	− 1.5
First order	SNF	$k_1 = 0.19$	0.42	116.60	− 0.14
($F = 100 \cdot e^{-k_1 t}$)	pH 7.4	$k_1 = 0.33$	0.72	110.66	0.6
Higuchi	SNF	$K_H = 21.95$	0.46	115.78	− 0.08
($F = k_H \cdot t^{0.5}$)	pH 7.4	$K_H = 26.24$	0.44	120.72	− 0.12
Korsmeyer-Peppas	SNF	$k_{KP} = 40.62$, n= 0.22	0.91	90.40	1.73
($F = k_{KP} \cdot t^n$)	pH 7.4	$k_{KP} = 48.82$, n= 0.22	0.92	93.94	1.79
Hixson-Crowell	SNF	$k_{HC} = 0.05$	0.2492	120.24	− 0.40
($F = 100 \cdot [1 - (1 - k_{HC} \cdot t)^3]$)	pH 7.4	$k_{HC} = 0.06$	0.4672	119.94	− 0.06
[a] Weibull	SNF	$\alpha = 1.67$, $\beta = 0.28$, $T_i = 0.24$	0.97	72.47	3.01
($F = 100 . [1 - e^{-(t-T1)\beta/\alpha}]$)	pH 7.4	$\alpha = 1.36$, $\beta = 0.36$, $T_i = 0.20$	0.98	72.62	3.32

[a]Best fit release kinetic model for free FVR. *F*: fraction (%) of drug released in time *t*; k_0: zero-order release constant; k_1: first-order release constant; k_H: Higuchi release constant; k_{KP}: release constant incorporating structural and geometric characteristics of the drug-dosage form; *n*: diffusional exponent indicating the drug release mechanism; α: scale parameter defining the time scale of the process; β is shape parameter characterizing the curve shape as exponential ($\beta = 1$), sigmoid or S-shaped, with upward curvature followed by a turning point ($\beta > 1$), or parabolic, with a higher initial slope and then consistent with an exponential ($\beta < 1$) [2]; T_i: location parameter representing the lag time before the onset of the dissolution or release process, which in most cases will be near zero; k_{HC} is the Hixon-Crowell release constant. The transport mechanism based on the "n" value of Korsmeyer-Peppas is described as: (1) $n \leq 0.5$, the drug diffusion from the polymer matrix corresponds to a Fickian diffusion or a quasi-Fickian diffusion mechanism. (2) $0.5 < n < 1$, an anomalous, non-Fickian drug diffusion occurs. (3) $n = 1$, a non-Fickian, case of II (relaxational) transport or zero-order release kinetics could be observed, and (4) $n > 1$, super case II transport was observed [3].

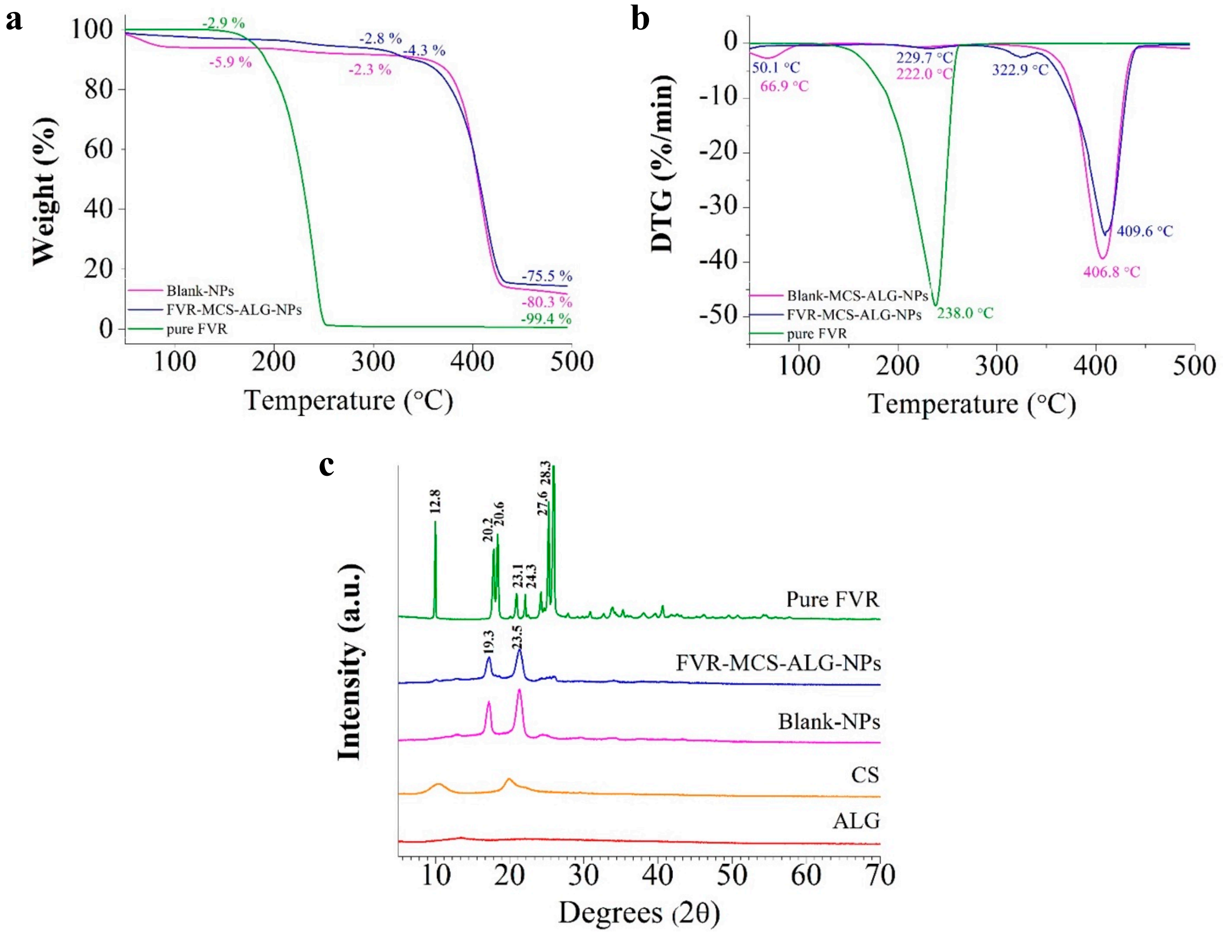

Figure S1. Characteristics of optimized FVR-MCS-ALG-NPs compared with the excipients: **(a)** TGA, **(b)** DTG thermal curves and **(c)** XRD pattern.

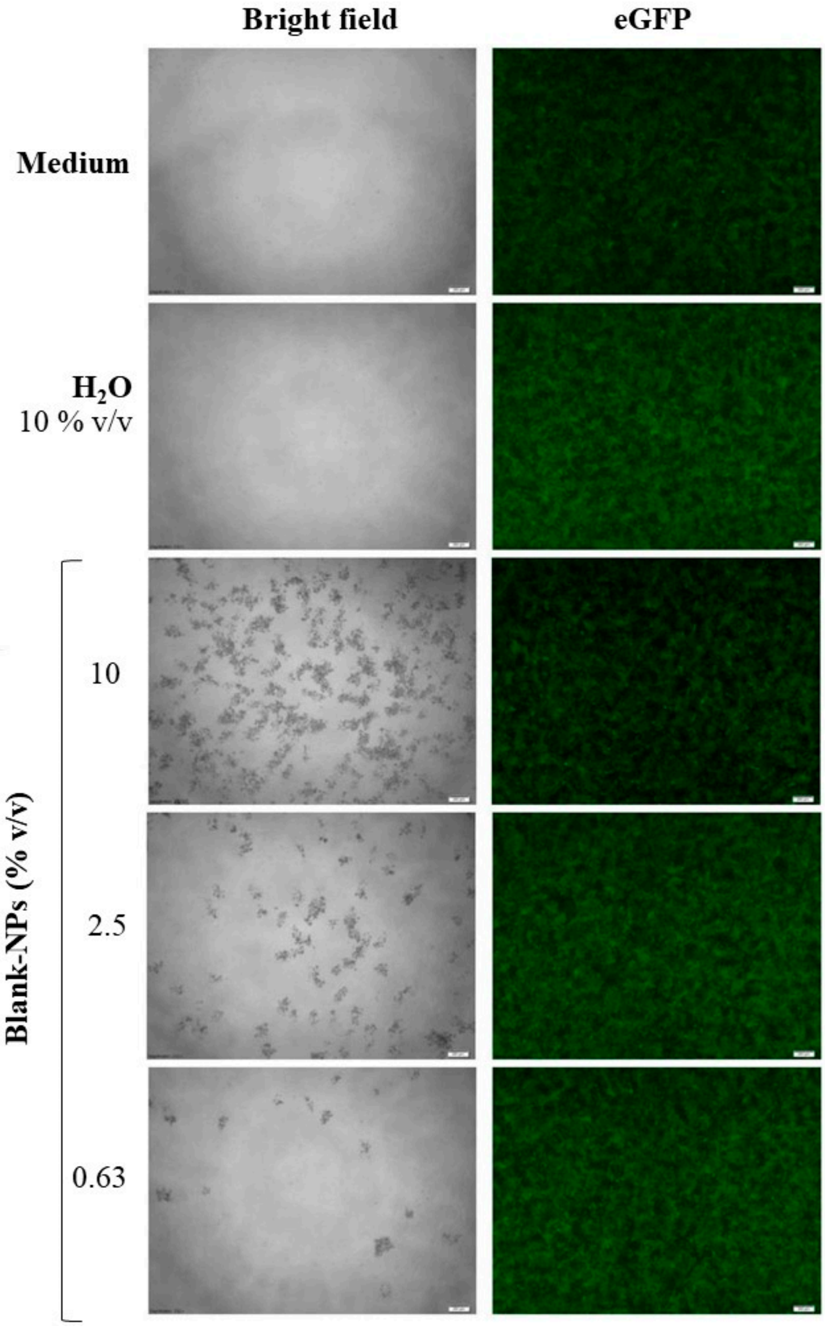

Figure S2. Cytotoxicity of the FVR-MCS-ALGP-NPs. eGFP-Vero cells were treated with H_2O or NPs at the indicated concentrations for 18 h. Control cells were the cells cultured in medium only. Cell viability was visualized by fluorescent microscopy and measured with a CCK-8 kit at an absorbance of 450 nm using a spectrophotometer.

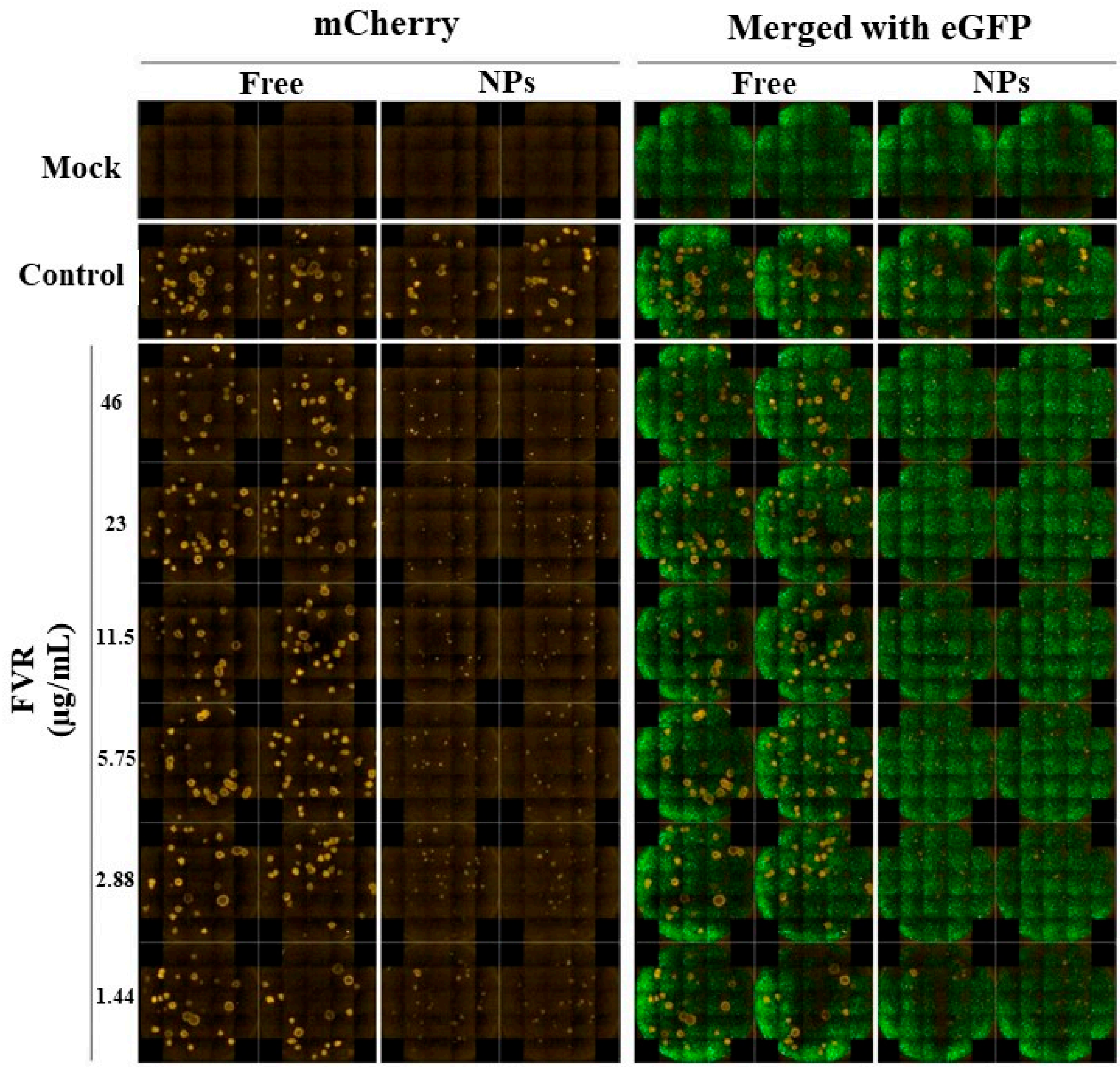

Figure S3. Antiviral effect of FVR (formulated and unformulated) from experiment 2. The stitched image of mCherry and eGFP signals from the cells were acquired by a high-content imaging system at 21 images/well. Each condition was tested in duplicate.

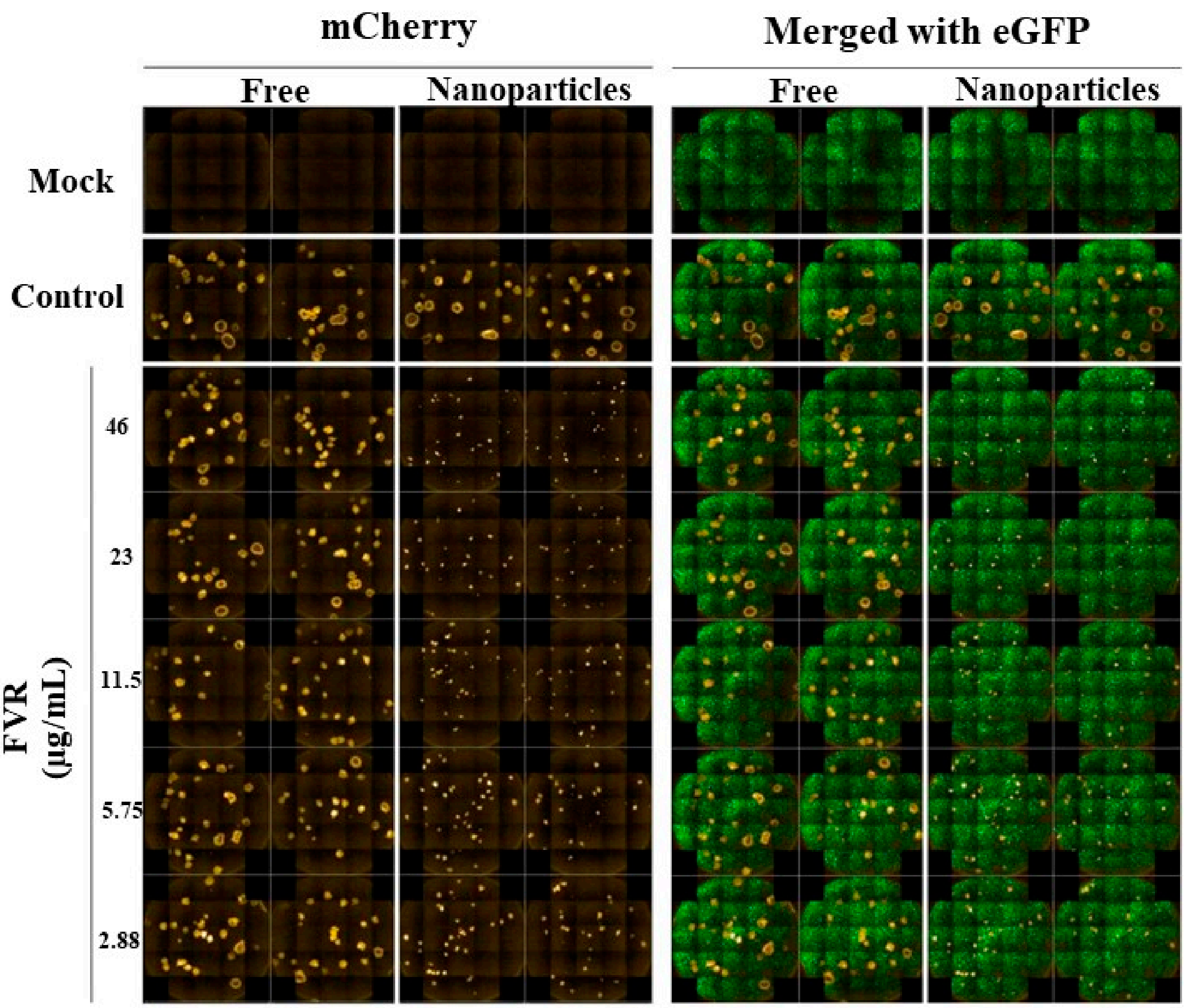

Figure S4. Antiviral effect of FVR (formulated and unformulated) from experiment 3. The stitched image of mCherry and eGFP signals from the cells were acquired by a high-content imaging system at 21 images/well. Each condition was tested in duplicate.

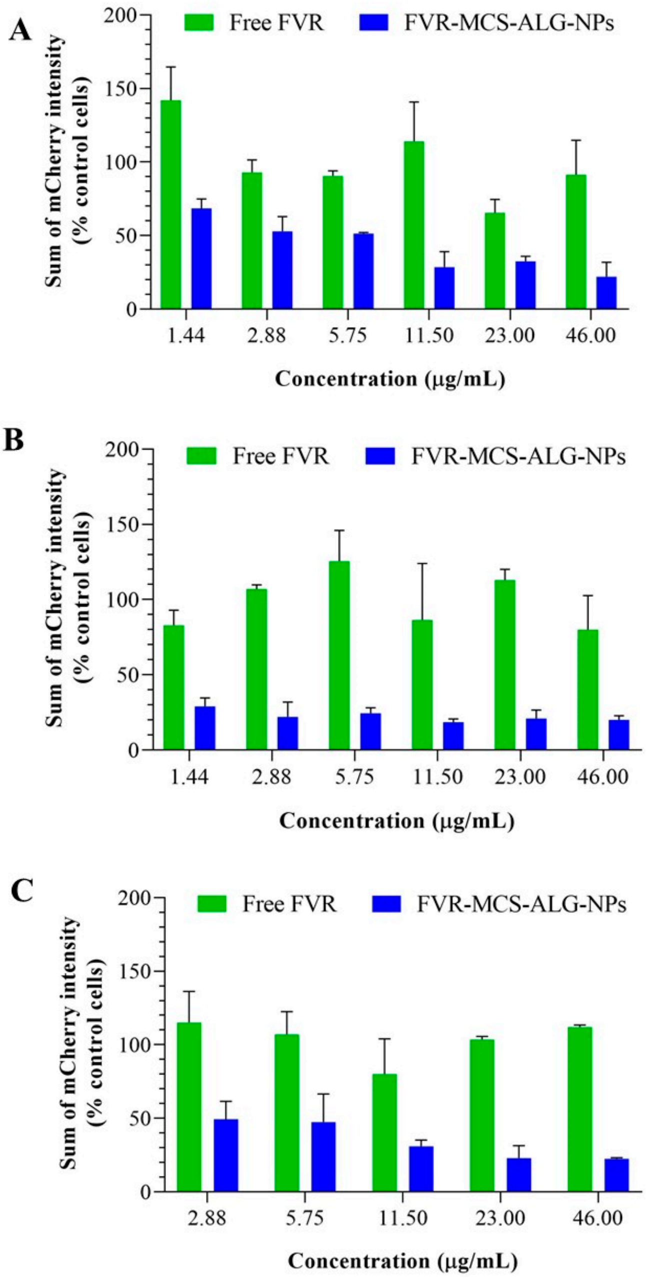

Figure S5. Quantitative data of the antiviral effect of FVR (formulated and unformulated) from 3 independent experiments. The mCherry fluorescent intensity of the infected cells treated with FVR from experiment 1 **(A)** (from Figure 7e), experiment 2 **(B)** (from Figure S3), and experiment 3 **(C)** (from Figure S4) was measured by the high-content imaging system. Data are shown as mean ± SD of the relative sum of mCherry fluorescent intensity (% control).

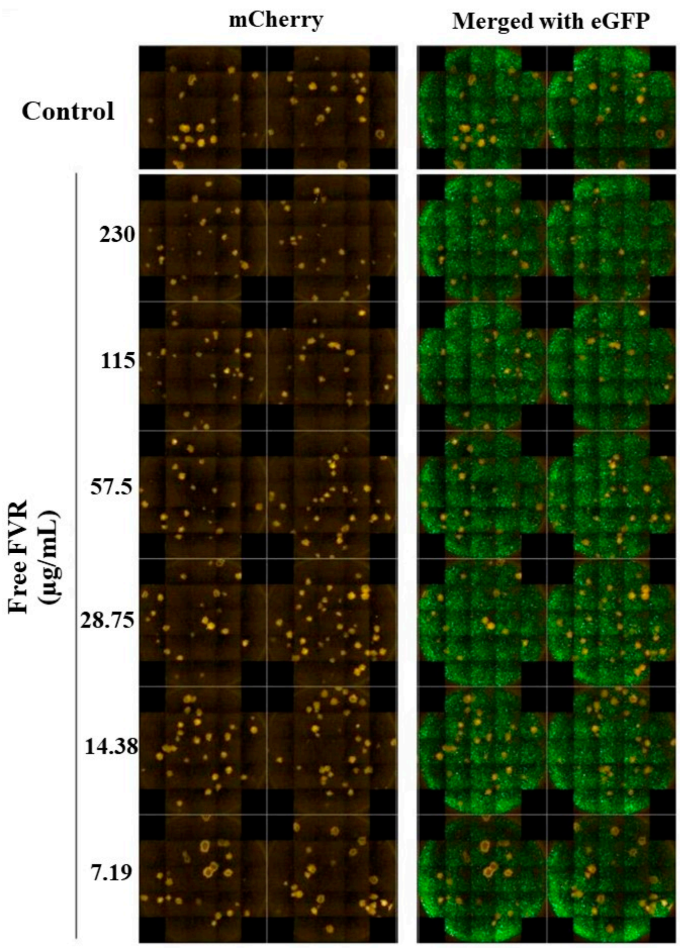

Figure S6. Antiviral effects of free FVR at high concentrations. The stitched images of the mCherry and eGFP signals from the cells were acquired by a high-content imaging system from the mCherry fluorescence intensity data in Figure 7d.

References

1. Jengarn, J., et al., *Genetic manipulation of porcine epidemic diarrhoea virus recovered from a full-length infectious cDNA clone.* J Gen Virol, 2015. **96**(8): p. 2206-2218.
2. Heredia, N.S., et al., *Comparative statistical analysis of the release kinetics models for nanoprecipitated drug delivery systems based on poly(lactic-co-glycolic acid).* PLoS One, 2022. **17**(3): p. e0264825.
3. Sorasitthiyanukarn, F.N., et al., *Chitosan/alginate nanoparticles as a promising carrier of novel curcumin diethyl diglutarate.* Int J Biol Macromol, 2019. **131**: p. 1125-1136.
